# Optimization of postoperative unicompartmental knee arthroplasty radiography using a phantom-based ray-summation positioning sheet

**DOI:** 10.1007/s12194-026-01049-7

**Published:** 2026-04-13

**Authors:** Kenta Katano, Kentaro Sakata, Madoka Nanao, Makoto Kudo, Tomohiro Sato, Toshikazu Imae, Kousaku Saotome, Yuichi Suzuki, Toshihiro Hayashi, Osamu Abe

**Affiliations:** 1 Radiology Center, The University of Tokyo Hospital, 7-3-1 Hongo, Bunkyo-ku, Tokyo, 113-8655 Japan; 2 Graduate School of Health Sciences, Ibaraki Prefectural University of Health Sciences, 4669-2 Ami, Inashiki-gun, Ami-machi, Ibaraki 300-0394 Japan

**Keywords:** Radiography, Radiation exposure reduction, Positioning errors, Technologist experience, Implant alignment evaluation

## Abstract

Achieving accurate positioning in postoperative unicompartmental knee arthroplasty (UKA) radiography is challenging, often necessitating increased exposure and examination time. We developed a positioning assistance sheet based on ray-summation images simulating rotation, flexion, and extension, and validated its efficacy in reducing exposure. We retrospectively analyzed 115 knees imaged between January 2024 and February 2025. A knee phantom was scanned to generate ray-summation images, which were used to design the assistance sheet. The mean number of exposures was compared before and after implementation, with a significant decrease from 3.26 to 2.37 (*P* = 0.03). The reduction was particularly pronounced among radiology technologists with < 5 years of experience. Furthermore, no significant differences were observed between left and right knees post-implementation (*P* = 0.30), confirming the sheet’s bilateral applicability. Consequently, the positioning assistance sheet significantly reduced the number of exposures required for postoperative UKA radiographs and proved effective regardless of technologist experience or laterality.

## Introduction

The incidence of knee osteoarthritis is rising alongside the aging population in Japan [1], establishing unicompartmental knee arthroplasty (UKA) as a primary surgical option for patients with isolated medial or lateral compartment disease. Compared with total knee arthroplasty, UKA offers distinct advantages, including accelerated postoperative recovery, preservation of knee kinematics, and higher patient satisfaction [2]. However, the long-term clinical success of UKA depends heavily on the accuracy of implant positioning and adequate postoperative follow-up [3].

**Fig. 1:**
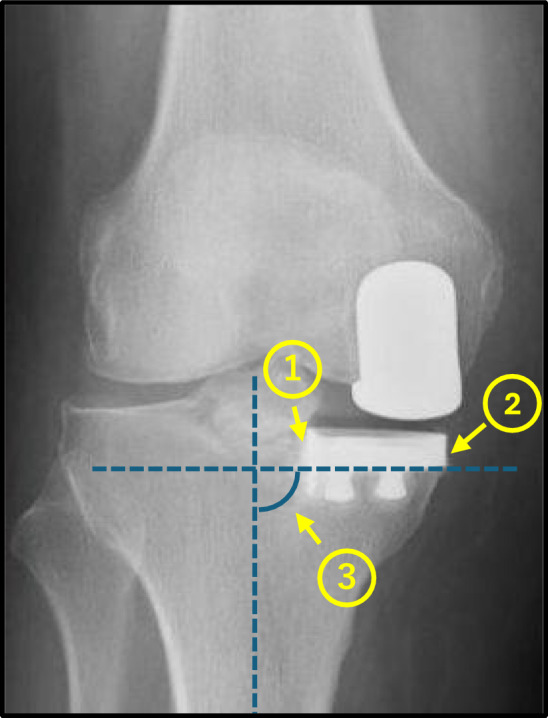
Clinical evaluation parameters on postoperative UKA X-rays: ① observation of loosening in the gap between the interbone eminence and the tibial baseplate, ② assessment of the positional relationship between the medial edge of the tibial baseplate and the medial edge of the tibia, ③ measurement of the tibial baseplate varus angle relative to the tibial axis

Conventional radiography remains the fundamental modality for the postoperative evaluation of UKA, providing critical information regarding implant position, alignment, and the presence of radiolucent lines or fractures. Because even subtle malalignment can induce early implant failure or persistent pain, obtaining highly reproducible radiographs is imperative. High reproducibility is particularly critical given that even minor rotational errors can introduce significant inaccuracies in component alignment measurements, most notably the coronal alignment of the tibial component—following UKA [4]. In clinical practice, precise radiographic evaluation of the tibial component is crucial for predicting long-term implant survival. For instance, as shown in Fig. 1, accurate assessment of component loosening, assessment of the positional relationship between the medial edge of the tibial baseplate and the medial edge of the tibial, and measurement of the tibial baseplate varus angle relative to the tibial axis requires a high-quality, reproducible anterior–posterior (AP) view. Suboptimal positioning can distort leading to incorrect clinical assessments of implant stability.

Nevertheless, achieving optimal positioning on postoperative radiographs is challenging due to several clinical factors. In the early postoperative phase, patients frequently experience significant pain and a restricted range of motion, making it difficult to maintain the necessary anatomical position for a true AP view. Furthermore, as follow-up examinations occur at multiple time points ranging from weeks to years after surgery, ensuring high reproducibility in knee positioning is extremely difficult. Such consistency is, however, indispensable for the accurate longitudinal assessment of implant alignment and the detection of subtle clinical changes. These issues extend to general radiography, wherein studies on digital image quality assurance have indicated that positioning errors account for the majority (approximately 80%) of rejected images [5]. The frequent need for retakes increases the number of exposures, resulting in elevated radiation doses and prolonged examination times-both of which are undesirable outcomes.

Although a previous study applied maximum-intensity projection analysis to lateral knee radiographs (without implants) to enhance the evaluation of anatomical structures [6], it did not reduce radiographic exposure. Furthermore, to the best of our knowledge, no study has specifically attempted to reduce the number of radiographic exposures following UKA.

Therefore, this study aimed to develop a positioning assistance sheet based on ray-summation images simulating knee rotation, flexion, and extension, and to evaluate its utility in reducing the number of exposures required for postoperative anteroposterior (AP) radiography following UKA, a procedure where retakes are notably frequent at our institution.

## Methods

### Study design and participants

This retrospective study was approved by the Institutional Review Board of the University of Tokyo Hospital (approval number: 11605-(7)). The study included a sample of 115 knees from 30 patients who underwent UKA between January 2024 and February 2025 (8 men and 22 women; mean age: 73.2 ± 14.8 years).

### Phantom construction

**Fig. 2:**
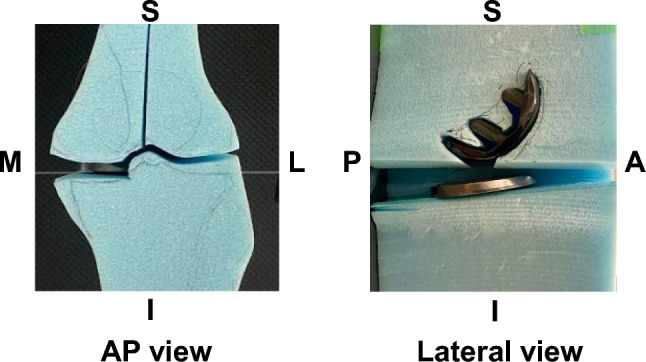
Knee phantom created by embedding a left medial UKA implant into high-density polystyrene, with the tibial baseplate positioned at a 5° posterior slope to reflect the standard surgical alignment. Directional arrows indicate the superior (S), inferior (I), medial (M), lateral (L), anterior (A), and posterior (P) directions

To construct a knee phantom simulating postoperative UKA, a UKA implant (JOURNEY II UK, Medial-left; Smith & Nephew, London, UK) was embedded in high-density polystyrene blocks. The femoral component was positioned with a posterior slope of 0°, whereas the tibial baseplate was implanted with a posterior slope of 5°, in accordance with previously reported optimal alignment parameters [7]. The femoral and tibial blocks were combined to form a left knee model (Fig. 2). Soft tissues, including muscle, fat, and skin, were not reproduced in this phantom, as the primary focus of this study was to evaluate implant positioning and radiographic reproducibility following UKA.

### Computed tomography (CT) imaging and ray-summation image generation

**Fig. 3:**
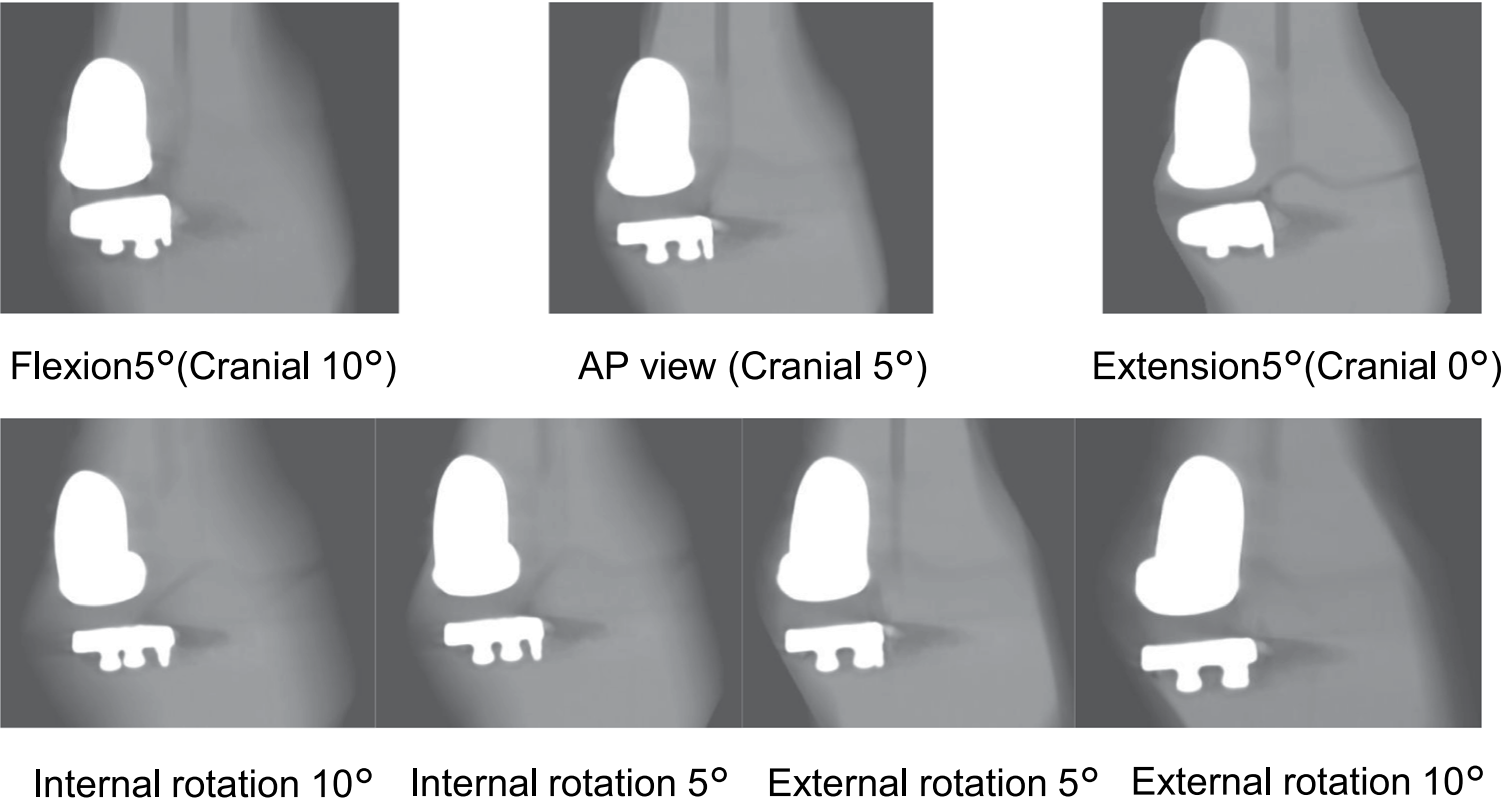
Positioning assistance sheet based on ray-summation images simulating variations in flexion, extension, and rotation, used to align the radiographic image of the UKA implant (tibial baseplate) with the central reference image to confirm a true anteroposterior (AP) view

The phantom was scanned using an 80-row CT scanner (Aquilion Prime; Canon Medical Systems, Otawara, Japan). Scan parameters were set as follows: tube voltage: 120 kVp; tube current: 200 mA; and pitch factor: 0.813. Images were reconstructed using a soft tissue kernel with a slice thickness and interval of 1.0 mm. Using this volumetric data, ray-summation images simulating various degrees of rotation, flexion, and extension were generated via a 3D image-processing workstation (Ziostation2; Ziosoft Co., Ltd., Tokyo, Japan). Ray-summation is a technique that calculates the sum of attenuation values of all voxels along a projection path, providing an image that closely mimics the contrast and overlapping structures seen in conventional X-rays. This differs from maximum-intensity projection (MIP), which extracts only the highest voxel value along the path and can result in the loss of depth and overlapping details. For this study, ray-summation was selected to provide a realistic reference for radiology technologists. The workstation allowed for the digital manipulation of the phantom’s orientation in 1-degree increments. This enabled the systematic generation of images across a wide range of rotation, flexion, and extension without the need for physical repositioning or multiple radiographic exposures of the actual phantom. These images served as the basis for the positioning assistance sheet design (Fig. 3).

### Positioning assistance sheet design and implementation

Prior to the implementation of the sheet, an institutional announcement was sent via email to all radiology technologists involved in UKA radiography to explain its purpose and usage. The positioning assistance sheet (Fig. 3) was printed on A4 paper, laminated for durability and hygiene, and strategically placed adjacent to the image-processing workstation (Console Advance; Fujifilm, Tokyo, Japan) in the radiography room. Implementation of the sheet began in November 2024, enabling radiology technologists to readily compare acquired images with the reference views provided on the sheet during examinations.

### Clinical validation

We compared the number of X-ray images taken before and after the introduction of the positioning aid sheet. The standard clinical protocol includes both AP and lateral images, but in this study, we focused on evaluating the number of exposures required to obtain a single clinically acceptable AP image. Additional exposures taken due to inadequate positioning were also included in the total analysis. Standard exposure settings for the knee AP view (both native and implanted) were 55 kVp, 5 mAs, and a source-to-image distance of 115 cm, without a grid or additional filtration. While these parameters served as a baseline, final settings were adjusted at the discretion of the radiology technologists based on individual patient physique. Although “pre-shot” techniques (tentative, low-dose exposures to confirm positioning) are sometimes employed, they were excluded from this analysis to strictly evaluate the efficacy of the positioning assistance sheet in reducing repeated diagnostic exposures.

Clinical validation was performed using UKA postoperative AP radiographs acquired through a digital radiography system (Ysio Digital Radiography System; Siemens Healthineers, Erlangen, Germany) equipped with a flat-panel detector (Calneo Smart C12; Fujifilm, Tokyo, Japan). Images were processed on an image-processing workstation (Console Advance; Fujifilm, Tokyo, Japan). All radiographs were performed in a single dedicated radiography room using the same imaging system. This ensured complete consistency in imaging geometry, detector characteristics, and post-processing algorithms throughout the study period.

**Fig. 4:**
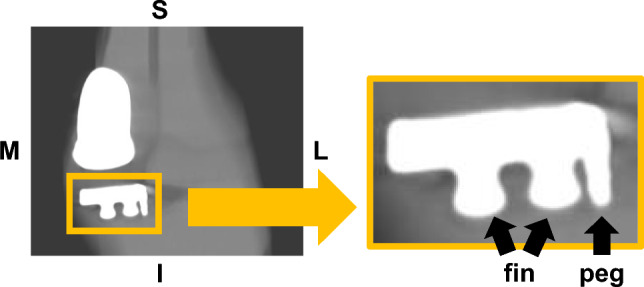
Ray-summation image and a magnified view illustrating the structural features of the tibial baseplate used for positioning, identifying the two medial structures as fins and the single lateral structure as the peg

The implants employed in this study featured three slender metallic structures extending distally from the tibial baseplate. For clarity, markers indicating the medial (M) and lateral (L) sides, as well as the superior (S) and inferior (I) directions, were added to the images. The two structures located on the medial side are referred to as “fins,” while the most lateral structure is referred to as the “peg” (Fig. 4).

The ideal AP view of the UKA implant model was defined based on two criteria to ensure accurate positioning. First, regarding rotational alignment (internal/external rotation), the tibial baseplate was required to appear horizontal without tilt, with the distance between the two fins being approximately twice that between the peg and the adjacent fin. Second, regarding sagittal alignment (flexion/extension), the image was considered an accurate AP view when the superior border of the tibial baseplate was parallel to the line connecting the inferior edges of the two fins and the peg.

### Statistical analysis

All statistical analyses were performed using Microsoft Excel (Microsoft Corp., Redmond, WA, USA). Before comparative analysis, the normality of the data distribution was assessed using the Shapiro–Wilk test. Because the data on the number of exposures exhibited an abnormal distribution (*P* < 0.05), nonparametric tests were conducted for all subsequent analyses. The Mann–Whitney U test was used to compare the overall mean number of exposures before and after the introduction of the positioning assistance sheet, as the comparison was performed between independent groups within the study period.

Additionally, the mean number of exposures was compared between radiology technologists with < 5 years of experience and those with ≥ 5 years using the Mann–Whitney U test to explore whether the effect of the sheet varied by experience level. A *P*-value < 0.05 was considered statistically significant. Because the positioning assistance sheet was designed using a left-sided implant model, confirming its clinical utility for right-sided examinations was essential. Ideally, laterality analysis (left vs. right) would compare the magnitude of reduction (pre-minus post-introduction values). However, owing to discrepancies in sample sizes between the pre- and post-introduction periods for each side (*n*_pre_  ≠  *n*_post_), a direct paired comparison of reduction magnitude was not feasible.Therefore, the Mann–Whitney *U* test was used to evaluate two aspects of laterality: differences in the mean number of exposures (1) between left and right knees in the pre- and post-introduction periods, and (2) between left and right knees within the post-introduction group. This approach aimed to confirm whether the positioning assistance sheet was equally effective for right-sided examinations. For all significance tests, a *P*-value < 0.05 was considered statistically significant.

## Results

### Overall comparison

**Fig. 5:**
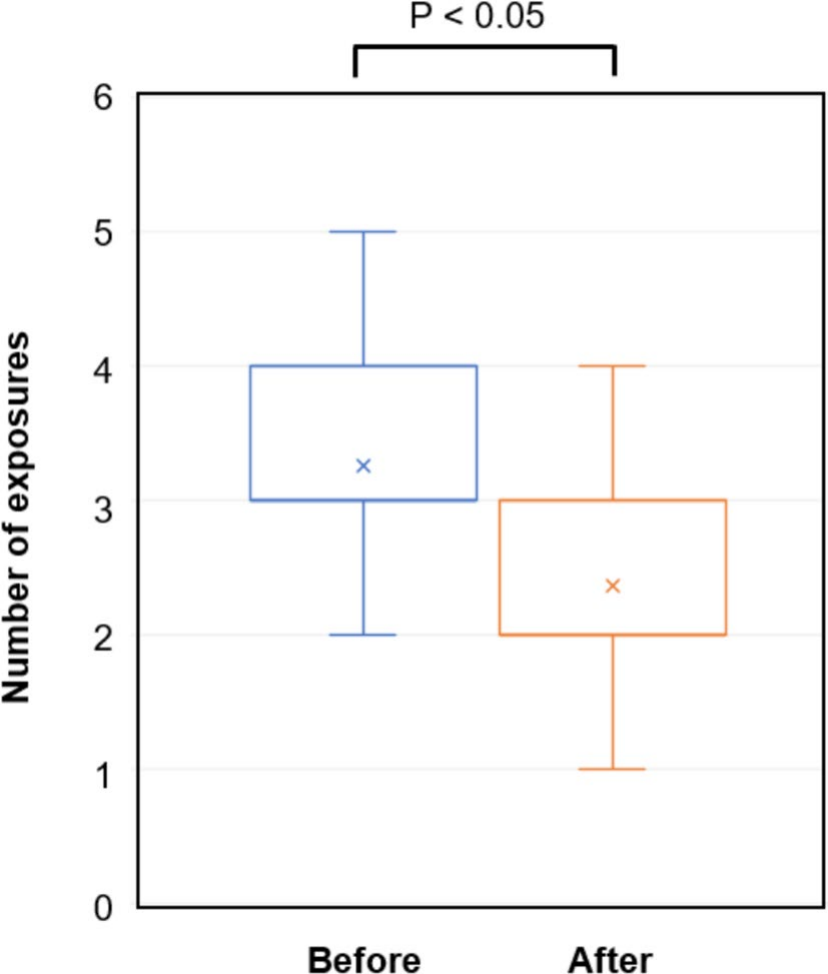
Boxplot showing the distribution of the average number of exposures before and after introduction of the positioning assistance sheet, with central markers (x) indicating mean values and a statistically significant reduction observed (P < 0.05)

A total of 115 knee images were analyzed, comprising 74 knees examined before and 41 knees examined after the introduction of the positioning assistance sheet. The overall mean number of exposures per examination decreased significantly from 3.26 before introduction to 2.37 after introduction, representing a mean reduction of approximately 0.89 (Wilcoxon signed-rank test, *P* = 0.03) (Fig. 5).

### Comparison by radiology technologist experience

**Fig. 6:**
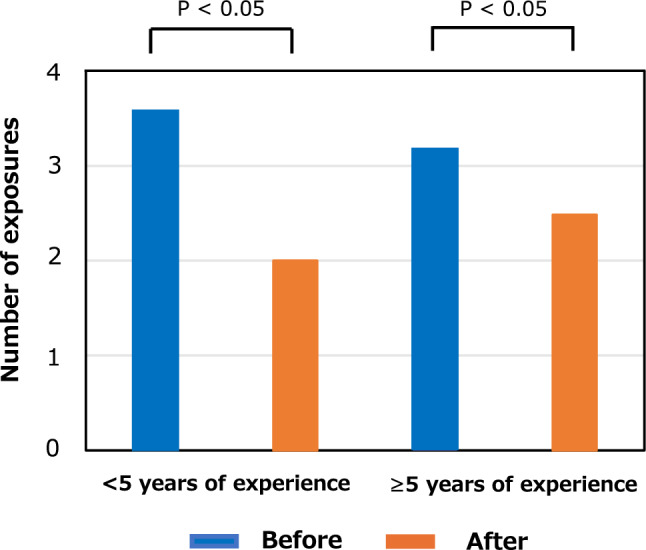
Bar chart illustrating the significant reduction (P < 0.05) in the mean number of exposures before and after sheet introduction, stratified by technologist experience (< 5 years vs. ≥ 5 years)

For radiology technologists with < 5 years of experience (*n* = 5), the mean number of exposures decreased from 3.54 before introduction to 2.00 after introduction (*P* = 0.02). Additionally, for those with ≥ 5 years of experience (*n* = 20), the mean number of exposures decreased from 3.16 to 2.48 (*P* = 0.03) (Fig. 6).

### Comparison between left and right knees

**Fig. 7:**
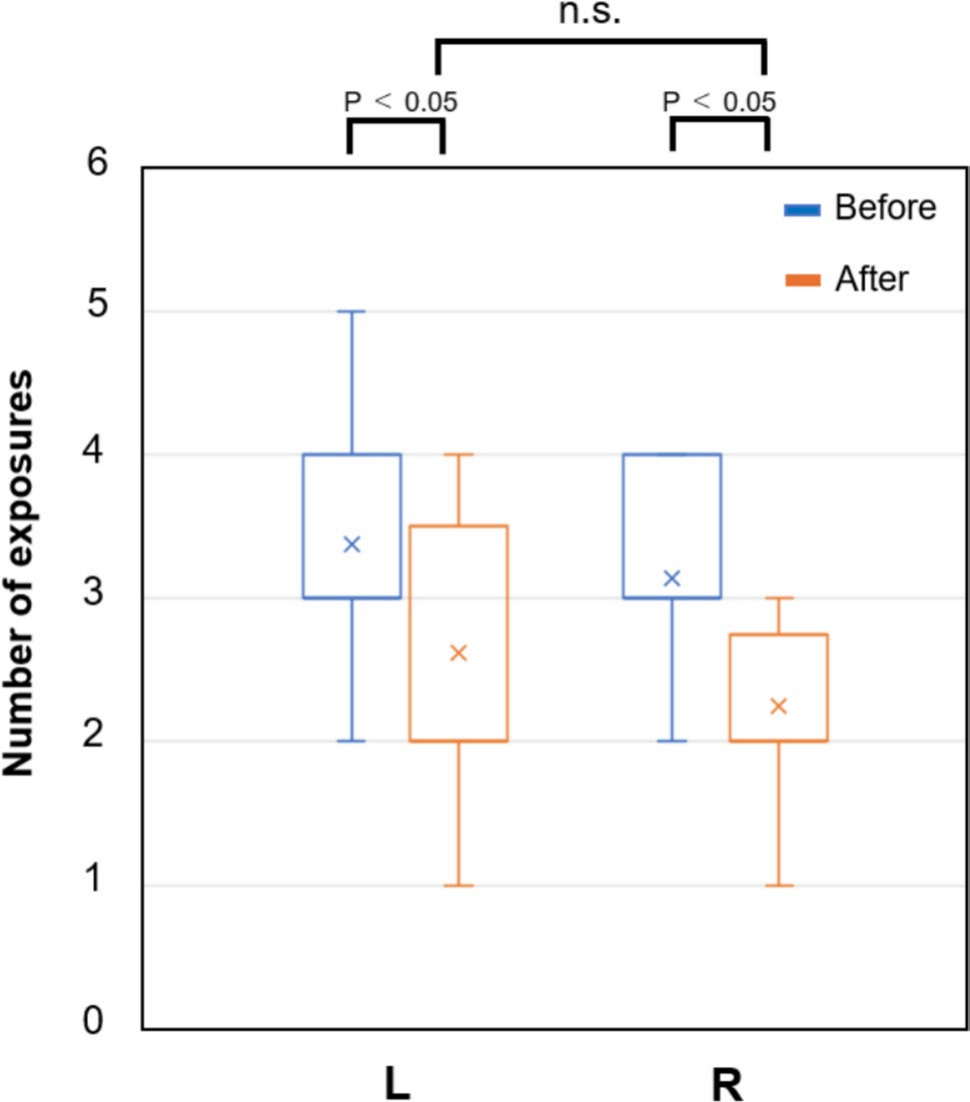
Boxplot comparing the number of exposures between left (L) and right (R) knees before and after sheet introduction, showing significant reductions on both sides with no significant difference between left and right knees in the post-introduction period

For left knees, the mean number of exposures decreased from 3.37 (before introduction) to 2.62 (after introduction), whereas for right knees, it decreased from 3.14 to 2.25. A significant reduction was observed on both sides (*P* = 0.00170 for left; *P* = 0.00169 for right). Furthermore, no significant difference was observed in the mean number of exposures between the left and right knees during the post-introduction period (*P* = 0.30) (Fig. 7).

## Discussion

This study demonstrated that the introduction of a positioning assistance sheet significantly reduces the number of exposures required for postoperative AP radiography following UKA. This reduction is clinically meaningful, as it directly contributes to lowering patient radiation dose and shortening examination times. To the best of our knowledge, no prior study has specifically proposed methods to decrease the number of exposures in AP radiography following UKA, highlighting the novelty of this investigation.

These results indicate that the positioning assistance sheet was used effectively during imaging procedures. Moreover, as each radiology technologist was responsible for an average of only three cases following the sheet’s introduction, it is unlikely that the observed reduction resulted from a natural improvement in individual technical skills over time. When stratified by experience, the reduction in exposures was more pronounced in the less experienced group (< 5 years). This finding suggests that the positioning assistance sheet may be particularly valuable for less-experienced radiology technologists by providing a reproducible reference for accurate positioning.

This study also confirmed the effectiveness of the positioning assistance sheet for both left and right knees. Specifically, the number of exposures decreased significantly in both groups following the sheet’s introduction. Although the sheet was designed using a left-sided implant model, no significant difference was observed in the mean number of exposures between the right and left knees during the post-introduction period. This finding strongly supports the conclusion that the sheet is equally applicable to examinations of both sides. A plausible explanation is that the essential positional relationship between the fins and peg of the tibial baseplate exhibits mirror symmetry, allowing the sheet’s visual guidance to remain effective regardless of laterality.

However, this study had several limitations that must be acknowledged. First, its retrospective, single-institution design restricted the generalizability of the results and prevented the complete exclusion of confounding factors, such as variations in radiology technologists experience or examination conditions over time. Second, owing to the discrepancy in the number of knees between the pre- and post-introduction periods (*n*_pre_  ≠  *n*_post_) for each side, an ideal paired comparison of the magnitude of exposure reduction could not be performed. Finally, the positioning assistance sheet was designed based on a specific medial left-sided implant model; therefore, its direct applicability to other UKA implant types, such as lateral models or those from different manufacturers, remains to be validated.

The findings of this study indicate that a simple, cost-effective tool, such as a positioning assistance sheet, can reduce retakes, improve efficiency, and enhance reproducibility in postoperative UKA radiography. However, there are some limitations to this study. First, we did not directly measure the total dose area product (DAP) or the exact examination time for each patient due to system-related data extraction constraints in this retrospective design. Although a reduction in the number of exposures inherently leads to lower radiation doses and shorter examination times, future prospective studies with larger sample sizes and the inclusion of these quantitative metrics are required to validate these findings.

## Conclusion

In this study, we developed a positioning assistance sheet based on phantom-derived ray-summation images. The clinical implementation of this sheet significantly reduced the number of exposures required for postoperative AP radiography following UKA. This reduction was particularly pronounced among less experienced radiology technologists, suggesting the sheet’s potential to compensate for limited clinical experience. Furthermore, although the sheet was designed using a left-sided model, it proved equally applicable to both left and right knees. These findings indicate that the positioning assistance sheet is a simple and effective tool for improving the efficiency and reproducibility of postoperative UKA radiography.

## Data Availability

The datasets generated and/or analyzed in this study are available from the corresponding author upon reasonable request.
